# Stomach contents of the archaeocete *Basilosaurus isis*: Apex predator in oceans of the late Eocene

**DOI:** 10.1371/journal.pone.0209021

**Published:** 2019-01-09

**Authors:** Manja Voss, Mohammed Sameh M. Antar, Iyad S. Zalmout, Philip D. Gingerich

**Affiliations:** 1 Museum für Naturkunde Berlin, Leibniz-Institute for Evolution and Biodiversity Science, Berlin, Germany; 2 Department of Geology and Paleontology, Nature Conservation Sector, Egyptian Environmental Affairs Agency, Cairo, Egypt; 3 Museum of Paleontology, University of Michigan, Ann Arbor, Michigan, United States of America; Liverpool John Moores University, UNITED KINGDOM

## Abstract

Apex predators live at the top of an ecological pyramid, preying on animals in the pyramid below and normally immune from predation themselves. Apex predators are often, but not always, the largest animals of their kind. The living killer whale *Orcinus orca* is an apex predator in modern world oceans. Here we focus on an earlier apex predator, the late Eocene archaeocete *Basilosaurus isis* from Wadi Al Hitan in Egypt, and show from stomach contents that it fed on smaller whales (juvenile *Dorudon atrox*) and large fishes (*Pycnodus mokattamensis*). Our observations, the first direct evidence of diet in *Basilosaurus isis*, confirm a predator-prey relationship of the two most frequently found fossil whales in Wadi Al-Hitan, *B*. *isis* and *D*. *atrox*. This extends our understanding of their paleoecology. Late Eocene *Basilosaurus isis*, late Miocene *Livyatan melvillei*, and modern *Orcinus orca* are three marine apex predators known from relatively short intervals of time. Little is known about whales as apex predators through much of the Cenozoic era, and whales as apex predators deserve more attention than they have received.

## Introduction

The modern killer whale *Orcinus orca* has a global distribution in the world’s oceans. Orcas, 6 to 8 meters in length, are apex (top) predators in the world’s oceans. They feed on a variety of invertebrate and vertebrate animals including squids, sharks, bony fishes, turtles, seabirds, and other marine mammals. Orca prey include at least 23 of the 89 or so other species of cetaceans [1, 2, 3, 4], which vary from 1.4 to 24 m in body length and span the full range of cetacean sizes. Orcas are known to attack, kill, and eat the largest of the toothed whales, the sperm whale *Physeter macrocephalus* [5], though sperm whales outweigh orcas by a factor of 10 (15 vs. 1.5 metric tonnes). To do this orcas cooperate and attack in groups [5, 6, 7]. In addition, orcas are known to attack and kill a variety of sharks, including the great white shark *Carcharodon carcharias* [8]. Orcas are reported to scavenge as well, but this is opportunistic and principally known from their scavenging of freshly killed mysticete carcasses attached to whaling ships [9].

Orca predation on whales was first reported by John Hunter in 1787 [10], who found the tail of a porpoise in the stomach of a ‘grampus’ (orca), showing, in Hunter’s words, that they “eat their own genus" [10]. In a more evocative study, Daniel Eschricht described removing the remains of 13 common porpoises and 14 seals, all in a “more or less digested state,” from the first and second stomachs of a decomposing orca [11]. Stomach contents provide definitive evidence of diet and *prima facie* if not definitive evidence of predation in orcas known to be predators.

**Fig 1:**
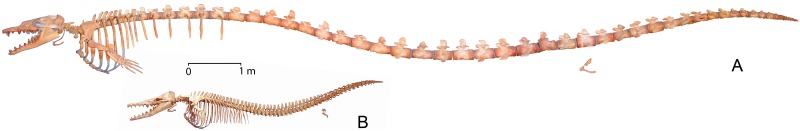
Skeletons of *Basilosaurus isis* (A; CGM 42195) and *Dorudon atrox* (B; CGM 42183 and UM 97512, 100146, 101215, 101222) from Wadi Al Hitan, Egypt, as exhibited at the University of Michigan. Both are adult, fully grown, and illustrated at the same scale (scale bar equals 1 meter). CGM 42195 shows a cast of a 15 meter long *B*. *isis* specimen.

The archaic whale *Basilosaurus* lived in the late Eocene epoch, from about 38–34 million years before present, and had a broad marine distribution at a time when few modern whales existed. Two species are known, *Basilosaurus isis* and its slightly younger sister taxon *B*. *cetoides*. Both were the largest whales of their time and dimorphic in size, with males having vertebrae and femora about 20% longer than those of females [12]. *Basilosaurus isis* (Fig 1), is early-to-middle Priabonian in age, or early late Eocene, and has a skeleton about 15–18 meters in length. *Basilosaurus cetoides*, not illustrated here, is middle-to-late Priabonian in age, or middle-to-late late Eocene, and has a skeleton about 17–20 meters in length [13]. Geographically, basilosaurids were widely distributed and possibly cosmopolitan in the late Eocene. The former species, *B*. *isis*, is found across North Africa from eastern Atlantic Ocean deposits in southern Morocco [14] to Tethys Sea deposits of Wadi Al Hitan and Qa’ Faydat ad Dahikiya in eastern Jordan [15]. The latter species, *B*. *cetoides*, is known from western Atlantic Ocean deposits bordering the Gulf of Mexico [13] to Tethys Sea deposits of the Qattara Depression in western Egypt [16].

**Fig 2:**
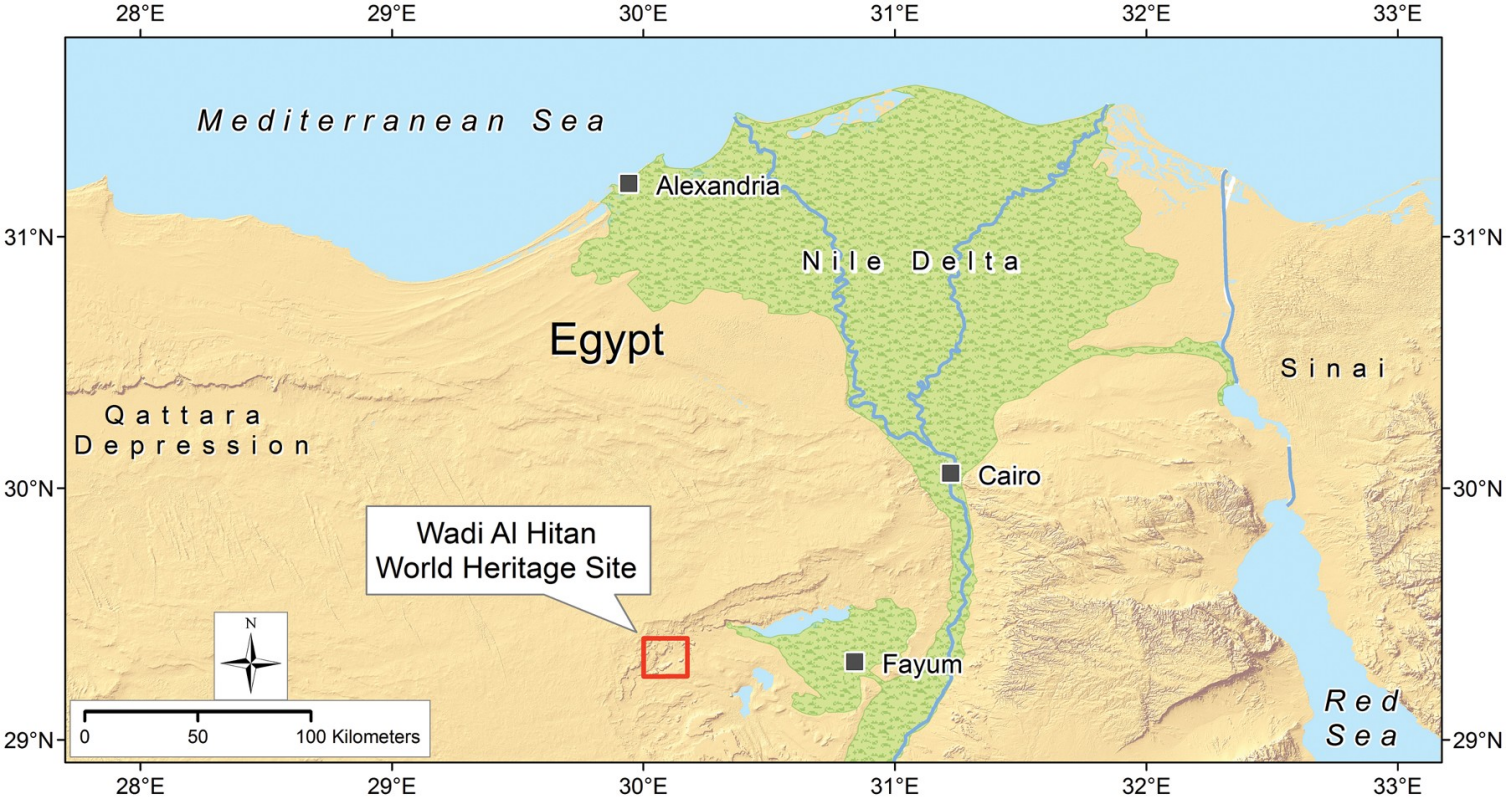
Wadi Al Hitan. Map showing the location of the Wadi Al Hitan UNESCO World Heritage Site in the Western Desert of Fayum Province, Egypt.

Stomach contents of *B*. *cetoides* were first reported to represent “fishes and sharks ranging up to approximately 50 cm in length,” prompting interpretation of *Basilosaurus* as an active predator catching, biting, and swallowing fishes “in a manner analogous to … the living killer whales” [17]. Here we describe the first stomach contents of *B*. *isis*. A new *B*. *isis* skeleton from Wadi Al Hitan (‘Valley of Whales’; Fig 2) in Egypt provides new clues to the diet of that species.

Wadi Al Hitan, 140 km southwest of Cairo, is a remarkable marine Eocene Konzentrat-Lagerstätte or fossil site with an exceptional abundance and concentration of fossils. The two most common whales, the 15–18 meter long archaeocete *Basilosaurus isis* and the 5-meter archaeocete *Dorudon atrox*, are each represented by hundreds of skeletons and partial skeletons. In addition, there are several less common archaeocete genera and species [18, 19, 20], three species of sirenians [21], two crocodilians [22], sea snakes [23], turtles [24, 25], bony fishes [26, 27], and many sharks [18, 28].

Three field observations concerning *Basilosaurus* and *Dorudon* are important for interpreting Wadi Al Hitan whales and their role in Eocene ecology:

- All of the *Basilosaurus isis* skeletons in Wadi Al Hitan have permanent teeth in place and vertebrae fully formed, meaning all are adult.
- Approximately one-half of the *Dorudon atrox* skeletons in Wadi Al Hitan have permanent teeth in place, and vertebrae fully formed—meaning they are adult; but the other half have deciduous teeth, and vertebrae with open growth plates—meaning they are juveniles. The juveniles include stages of ontogeny from neonatal to subadult.
- Several of the subadult *Dorudon atrox* skulls in Wadi Al Hitan have bite marks on the skull roof.

These observations led to the idea that the late Eocene shallow sea covering what is now Wadi Al Hitan was a calving area for *Dorudon*, and, related to this, a feeding area for predatory *Basilosaurus* [18].

Evidence of whale-on-whale predation in the Eocene is limited, but this has grown over time with new information from Wadi Al Hitan. *Dorudon* skulls with bite marks were described and illustrated by Uhen [29], and further studied by Fahlke [30]. Both found the bite marks to be consistent with predation by *Basilosaurus*. Here we augment this finding by describing remains of juvenile *Dorudon atrox* and remains of large fish found as stomach contents in a Wadi Al Hitan skeleton of *Basilosaurus isis*.

## Materials and methods

The Wadi Al Hitan *Basilosaurus isis* specimen with stomach contents described here is known by its map number, WH 10001. The skeleton as a whole does not have a museum number because most parts of the skeleton were not collected. WH 10001 was found in December, 2010, in the eastern part of the Wadi Al Hitan protected area, north of Garet Gehannam, on a broad open plain of eroding glauconitic siltstone and shale of the Gehannam Formation. The desert here has no cover of vegetation and is largely wind-eroded. Geographic coordinates of WH 10001 are 29.36030° N and 30.15621° E. Cetacean and sirenian specimens in this area are found as concentrations of bones representing isolated individual skeletons. Some skeletons remain articulated or partially articulated, while others have been disturbed by scavengers or ocean currents. Bones of the WH 10001 skeleton are disarticulated and scattered over a small area. All are clearly associated, and the vertebrate specimen closest to WH 10001 is a partial skeleton of a sirenian that was found 150 meters away.

The WH 10001 *Basilosaurus* skeleton lies on greenish-yellow silty clay with occasional small bivalves. Bones in the middle of the skeleton are covered by a thin layer of glauconitic sand with many small shark teeth. The silty clay with the *Basilosaurus* skeleton is overlain by a glauconite-rich shale that has been largely removed by erosion.

The *Basilosaurus* locality and the entire open plain surrounding it are in the lower part of the Gehannam Formation, deposited during sea level rise following the Pr-1 low sea stand [31]. The Pr-1 low sea stand marks the Bartonian–Priabonian stage/age boundary marking the transition from the middle to the late Eocene. The closest stratigraphic sections are Amin Strougo’s sections S329 and S340 some 5 km south of WH 10001, where the lower Gehannam Formation is in nannoplankton zone NP17 and in planktonic foraminiferal zone P14 [32]. Combining this evidence, the age of WH 10001 is approximately 37.5 Ma [33].

WH 10001 was found during a mapping project initiated to improve understanding of the preservation and taphonomy of Wadi Al Hitan cetacean and sirenian skeletons. The WH 10001 *Basilosaurus* skeleton was exposed on the surface and chosen for further excavation. During excavation we found the remains of sharks, a nice prearticular dentition of the large bony fish *Pycnodus mokattamensis*, and numerous subadult remains of the smaller basilosaurid archaeocete *Dorudon atrox*, all mixed with bones of the skeleton of the larger *Basilosaurus isis*. Specimens interpreted as possible stomach contents were collected and, following preparation, assigned Cairo Geological Museum numbers CGM 60552 through 60580. The *Basilosaurus* skeleton itself remains at the site, where it was covered with sediment to preserve it.

**Fig 3:**
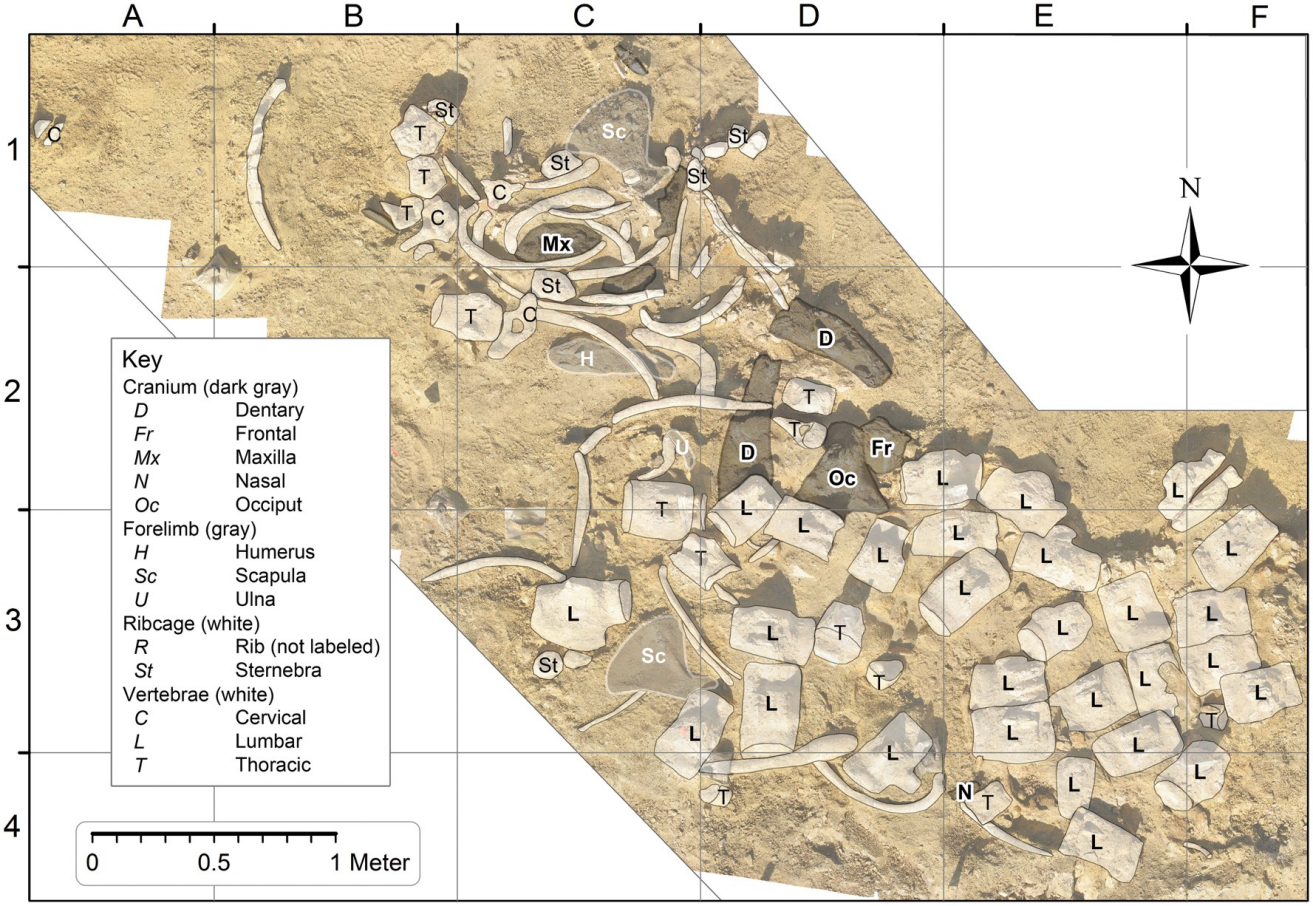
Photomosaic of *Basilosaurus isis* WH 10001 from the Gehannam Formation of Wadi Al Hitan. *B*. *isis* skeletons in the overlying Birket Qarun Formation are often partially to fully articulated, but here in bones are disarticulated and scattered. Note that thoracic vertebrae, ribs, sternebrae, and forelimb elements predominate in the northwestern quadrant of the map, whereas lumbar vertebrae are concentrated in the southeastern quadrant. Most cranial elements (dark gray) are near the center, but *B*. *isis* teeth are distributed more widely (Fig 4). Disarticulation and scatter observed here suggest disturbance by scavengers and possibly long exposure on the sea floor.

As a basis for Fig 3 (and all other figures built upon Fig 3), a map of the excavation site was constructed by laying out an orthographic 4-meter (N–S) by 6-meter (E–W) grid over the *Basilosaurus* skeleton as it lay in the field, and then taking multiple overlapping photographs of the contents of each grid cell. Meter-square grid cells are identified by their columns (A–F) and rows (1–4), with bones being distributed in cells from A1 through F4 (Table 1). Grid cells in the northeast part of the grid, downslope from the skeleton, did not contain bones because the stratigraphic level is too low. Grid cells in the southwest part of the grid seem to be beyond the margin of the skeleton, but it is possible that a few skeletal elements remain there covered by sediment. The glauconitic sandstone is well-cemented, making it difficult to excavate when unweathered.

**Table 1:** Elements recovered from the WH 10001 *Basilosaurus isis* excavation in Wadi Al-Hitan, Egypt.

Species	Grid cell	CGM specimen	UM cast	Description	Illustration
*Basilosaurus isis*	2B	60552	118142	Left permanent molar M^1^	Fig 5A
*Basilosaurus isis*	2D	60554	118144	Left or right permanent premolar P_4_ or P^4^	Fig 5E
*Basilosaurus isis*	2D	60555	118145	Piece of rib	Fig 9B
*Basilosaurus isis*	2D	60558	118148	Right permanent molar M_2_	Fig 5D
*Basilosaurus isis*	3C	60561	118151	Right permanent incisor I_2_ or I_3_	Fig 5G
*Basilosaurus isis*	3E	60565	118155	Right permanent incisor I_1_ without enamel	Fig 5H
*Basilosaurus isis*	3F	60568	118158	Right permanent incisor I^1^	Fig 5C
*Basilosaurus isis*	4E	60570	118160	Posterior half of right permanent premolar P_4_	Fig 5B
*Basilosaurus isis*	4E	60572	118162	Piece of rib	—
*Basilosaurus isis*	4E	60575	118165	Rostral part of a left or right nasal	—
*Basilosaurus isis*	4F	60579	118169	Right permanent canine C_1_	Fig 5F
*Dorudon atrox*	3C	60560	118150	Vertebral centrum (T17?)	Fig 8D
*Dorudon atrox*	2C	60553	118143	Vertebral centrum	Fig 8A
*Dorudon atrox*	2C	60556	118146	Vertebral epiphysis	Fig 8F
*Dorudon atrox*	2C	60576	118166	Rib fragment	Fig 8H
*Dorudon atrox*	2D	60559	118149	Vertebral centrum (T16?)	Fig 8C
*Dorudon atrox*	3C	60562	118152	Supraorbital process of right frontal	Fig 7C
*Dorudon atrox*	3C/D	60563	118153	Rib pieces	—
*Dorudon atrox*	3D	60571	118161	Left rib	Fig 8G
*Dorudon atrox*	3E	60566	118156	Vertebral centrum (?)	Fig 8E
*Dorudon atrox*	3E/F	60567	118157	Right dentary with dP_3-4_ and M_1_ erupting	Fig 7A
*Dorudon atrox*	4D	60569	118159	Supraorbital process of right frontal	Fig 7D
*Dorudon atrox*	4E	60574	118164	Left tympanic bulla	Fig 7F
*Dorudon atrox*	4E	60577	118167	Left squamosal	Fig 7E
*Dorudon atrox*	4E	60578	118168	Vertebral centrum (T14 or 15?)	Fig 8B
*Pycnodus mokattamensis*	—	60580	118170	Isolated tooth	—
*Pycnodus mokattamensis*	3D	60564	118154	Prearticular with teeth	Fig 9A
*Carcharocles sokolowi*	2C	60557	118147	Shark tooth	Fig 9B
Indet.	4E	60573	118163	Mass of bone	—

Specimens are sorted by taxon and by the grid cell where each was found on the map in Fig 3 (and all other figures built upon Fig 3).

Photographs of all grid cells and their contents were stitched together using Agisoft PhotoScan Professional software. The resulting composite was then drawn and interpreted by co-author PG using ESRI ArcMap software. The map underlying Fig 2 was compiled and drawn using ArcGIS software by co-author PG.

*Dorudon atrox* specimens interpreted as stomach contents were identified by comparison to *D*. *atrox* specimens representing a range of ontogenetic stages [29]. Elements of the WH 10001 skeleton of *Basilosaurus isis* were identified by comparison to CGM 42195 (Fig 1), UM 93231 (WH-30), and UM 97526 (WH-148), which are, respectively, a nearly complete skeleton, much of the anterior part of a second skeleton, and the posterior part of a third skeleton of *B*. *isis*, all collected from the overlying and slightly younger Birket Qarun Formation of Wadi Al Hitan.

## Results

### Basilosaurus isis

A photomosaic of the WH 10001 *Basilosaurus isis* skeleton as it lies in the field is illustrated in Fig 3. Late Eocene *B*. *isis* skeletons in the overlying Birket Qarun Formation are often partially to fully articulated, but here in the Gehannam Formation bones of WH 10001 are disarticulated and somewhat scattered. CGM 42195 and UM 97526 show that the vertebral formula of *B*. *isis* was 7:16:19:4:20, representing counts of cervical, thoracic, lumbar, sacral, and caudal vertebrae, for a total count of 66 vertebrae. In the field we identified 4 cervicals, 10 thoracics, 28 ‘lumbars,’ no sacrals, and no caudals preserved in WH 10001 (but see comment below). Some cervical and thoracic vertebrae of WH 10001 are missing from the site, as are all of the smaller caudals. It appears that much of the caudal part of the skeleton weathered out and was lost to erosion before the cranial part of the skeleton emerged.

Posterior thoracics resemble lumbars in size and proportions, but on close examination thoracics can be identified by the presence of facets for rib articulations. Lumbar, sacral, and anterior caudal vertebrae can only be distinguished and identified reliably to position when they are found in sequence, which is not the case here. Thus our count of 28 ‘lumbar’ vertebrae for WH 10001 no doubt includes a number of posterior thoracics, sacrals, and anterior caudals. It was not possible to identify all of the vertebrae of WH 10001 reliably in situ in the field. However, we can say that the WH 10001 *Basilosaurus* is an adult skeleton based on co-ossification of most vertebral epiphyses and based on tooth wear of the permanent teeth (see below).

The longest *Basilosaurus isis* vertebrae in the WH 10001 excavation are lumbars that are 350–360 mm in length. For comparison, the longest lumbar vertebrae in the CGM 42195 adult skeleton of *B*. *isis* are 330 mm long, and both lumbar length and femur length indicate that the CGM 42195 skeleton is female. The longest vertebrae in the UM 93231 adult skeleton of *B*. *isis* are 355 mm in length, and both lumbar length and femur length indicate that this skeleton is male. We infer from lumbar length that the WH 10001 adult skeleton of *B*. *isis* is also male.

#### Taphonomy of *Basilosaurus isis*

One key to understanding the taphonomy of vertebrates is to evaluate the quantity and quality of the specimens found [34]. Much information can be deduced from the completeness, disarticulation, degree of scattering and relative positions, and from modifications of the skeletal elements [35]. Minimum convex polygons (not shown) were constructed to enclose elements representing different parts of the *B*. *isis* skeleton. These show that the skeleton is relatively complete, but also completely disarticulated. We assessed the completeness of WH 10001, following [29], by calculating the proportion of bones of the skeleton represented at the site. Our excavation was carried out at the size scale of the skull, vertebrae, ribs, and long bones of the limbs. The sediment was well indurated, and a search for smaller hand and foot bones was beyond what we could attempt. Based on the skull, vertebrae, ribs, and long bones of the limbs, we estimate that the WH 10001 skeleton is 77/115 or 67% complete.

Cranial bones and the dentaries of WH 10001 were concentrated in the north center of the excavation, but one broken nasal was found near the southern margin. Forelimb bones were found in the northwestern quadrant of the excavation, but one scapula was found in the southwestern quadrant. All four of the expected seven cervical vertebrae identified in WH 10001 were found in the northwestern quadrant of the excavation. Most thoracic vertebrae, ribs, and sternebrae, which together formed the thorax of WH 10001 in life, were found in the northern half of the excavation. However, there are a few thoracics and ribs scattered as far as the southern edge of the excavation, and there is one sternebra in the southwestern quadrant.

Vertebrae identified as lumbars are limited to, and concentrated in, the southern half of the map. These are the largest vertebrae of the skeleton and were connected to each other in life by fibrocartilage of the successive intervertebral disks. Observation of many skeletons in the field indicates that vertebrae of the lumbar series of *Basilosaurus* are usually the last to separate when connective tissue decomposes, and their large size means they are less likely to be moved by ocean currents or by scavengers feeding on the decomposing body. The lumbars of WH 10001 are completely disarticulated, but they remain close to each other in a relatively small area.

Bones of WH 10001 were clearly moved on the sea floor, but all were found in a restricted area. This is typical for a whale-fall assemblage [36], where a carcass falls slowly to the sea floor in relatively quiet water. Whale skeletons have a particular bauplan and taphonomic predisposition, which leads to a characteristic pattern and sequence of disarticulation of skeletal elements [37, 38]. Because of their length, *Basilosaurus* carcasses characteristically produce an s-shaped arrangement of vertebrae articulated on the sea floor, which may be modified, up to complete disarticulation, depending on exposure time, scavenging, and water currents [39]. WH-10001 shows that most bones are no longer located in anatomically correct position along the anterior-posterior-axis, and yet their former positions can be roughly traced. WH 10001 also shows that none of the bones remain in articulation.

The combination of high completeness and great disarticulation suggests that WH 10001 fell to the sea floor as a complete carcass (possibly missing distal limbs, which may have been scavenged before the descent). The carcass was then destabilized by an accumulation of gases in the abdominal region, a breakdown of connective tissue, and disturbance by scavengers before burial. Burial was slow and took place in a sheltered environment maintaining close spatial proximity of the bones. This accords well with deposition of the carcass in a sediment-starved offshore environment at Wadi Al Hitan and with the interpretation of the enclosing greenish glauconitic sand as a condensed horizon [39].

We interpret the disarticulation and scatter of bones observed in WH 10001 as indicating disturbance by scavengers and movement by water currents during a relatively long time of exposure on the sea floor. Extant cetaceans have bones that are less dense than those of land mammals [40] but Eocene archaeocetes have bones retaining the density of land mammals [41, 42]. Decay, microbial activity, and bone diagenesis can also affect density [36, 43]. Higher density decreases and lower density increases the chance of bones being moved by currents on the sea floor.

Concentrations of elements representing different parts of the skeleton are evidence of the original resting place for each part. The cranium and dentaries probably came to rest in what is now the north center of the excavation, cervicals are concentrated to the north and west of the cranium, thoracics, ribs, sternebrae, and forelimb elements are concentrated west of center, and lumbars are concentrated south and east of center. We envision a skeleton that came to rest with the head near the center, and the cervix, thorax and forelimbs, lumbus, and tail arrayed in a counter-clockwise spiral relative to the head, a spiral that expanded away from the head.

We attribute the scatter of elements relative to their original resting place to scavengers, probably principally sharks both small and large (see below). However, the exceptions are also interesting. One cranial element, a broken nasal, was found near the southern margin of the excavation. Several smaller thoracics, some large and small ribs, one sternebra, and the right scapula were found south and east of most of their counterparts. We postulate a bottom current flowing from northwest to southeast to explain these unidirectional displacements.

The duration of exposure of the WH 10001 skeleton on the sea floor is difficult to estimate. As indicated above, disarticulation, movement of bones, and deposition on a glauconitic sand favor long exposure, but the paucity of epibionts (oysters, barnacles, bryozoans, etc.) suggests more limited exposure.

#### Teeth of *Basilosaurus isis*

**Fig 4:**
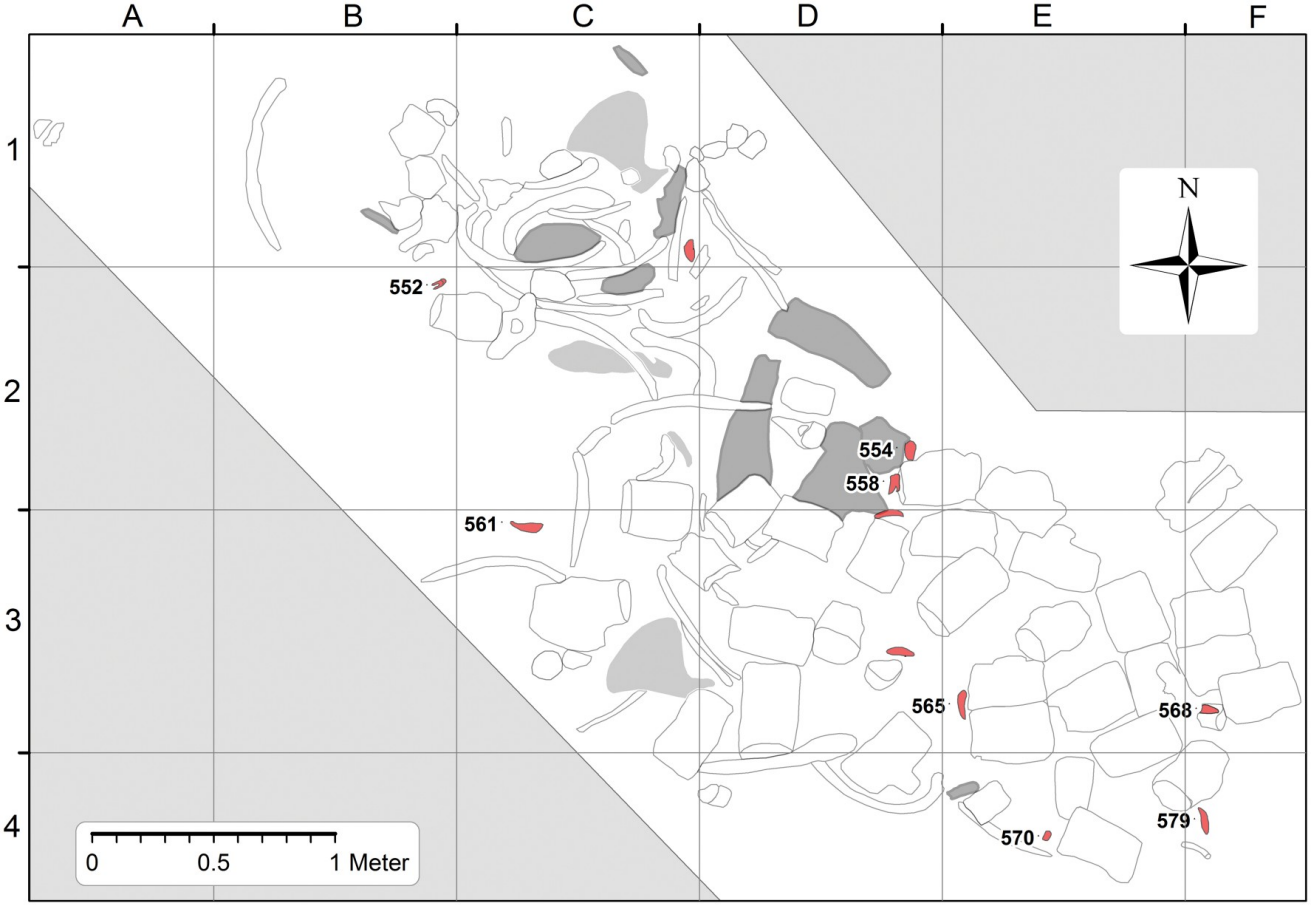
Map of *Basilosaurus isis* WH 10001 with locations of 13 permanent teeth superimposed. The eight *B*. *isis* teeth studied here, illustrated in Fig 5, are identified by the last three digits of their CGM museum number (CGM 60552, etc.). Outlines of *B*. *isis* cranial remains are filled with darker shading, and outlines of *B*. *isis* forelimb elements are filled with lighter shading.

The distribution of *Basilosaurus* teeth is illustrated in Fig 4, where 11 teeth are mapped. Five teeth are in or near the concentration of *Basilosaurus* cranial bones, and the rest are south and east of this, which is consistent with movement by the northwest-to-southeast bottom current postulated here.

**Fig 5:**
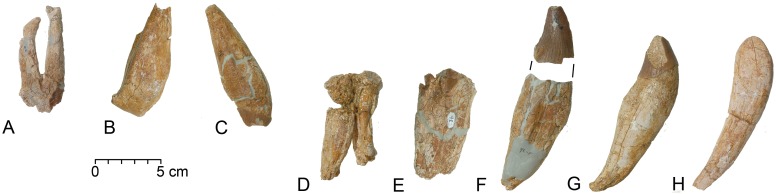
Permanent teeth of adult *Basilosaurus isis* WH 10001. The position of each tooth in the WH 10001 *B*. *isis* excavation is shown in Fig 4, identified by the last three digits of its CGM number. A, CGM 60552, left M^1^ or M^2^. B, CGM 60570, anterior half of right P^4^ or posterior half of left P_4_. C, CGM 60568, right I^1^. D, CGM 60558, right M_2_. E, CGM 60554, posterior half of right P_4_. F, CGM 60579, left C_1_. G, CGM 60561, right I_2_. H, CGM 60565, right I_1_. Note heavy wear, especially on premolars, consistent with predation on large animals and relative maturity of the WH 10001 *B*. *isis* itself. Abbreviations: *C*_*1*_, first lower canine; *I1*, first upper incisor; *I*_*1-2*_, lower incisor 1–2; *M*_*2*_, lower molar 2; *M*^*1*-2^, upper molar 1–2; *P*_*4*_, lower premolar 4; *P*^*4*^, upper premolar 4.

The eight *Basilosaurus* teeth that we were able to recover, prepare, and identify are illustrated in Fig 5. These are labeled on the map in Fig 4 using the last three digits of their CGM museum number. Three of the eight are regarded as upper teeth and five are regarded as lower teeth. All were identified by comparison with the virtually complete upper and lower dentition of *B*. *isis* in CGM 42195. Measurements are given in Table 2.

**Table 2:** Measurements of teeth recovered from the WH 10001 *Basilosaurus isis* excavation in Wadi Al-Hitan, Egypt.

Species	Grid cell	CGM specimen	Description	Anteroposterior length (mm)	Transverse width (mm)	Illustration
*Basilosaurus isis*	2B	60552	Upper molar	34	17	Fig 5A
*Basilosaurus isis*	2D	60554	Right P_4_	—	28	Fig 5E
*Basilosaurus isis*	2D	60558	Lower molar	47	19	Fig 5D
*Basilosaurus isis*	3C	60561	Right I_x_	31	21	Fig 5G
*Basilosaurus isis*	3E	60565	Right I_1_	—	—	Fig 5H
*Basilosaurus isis*	3F	60568	Right I^1^	—	—	Fig 5C
*Basilosaurus isis*	4E	60570	Premolar	—	—	Fig 5B
*Basilosaurus isis*	4E/F	60579	Left C_1_	36*	25*	Fig 5F
*Dorudon atrox*	3E/F	60567	dP_4_	56	—	Fig 7A

Specimens are sorted by species and specimen number.

*Measurements with asterisks are estimated.

In the upper dentition, CGM 60552 from grid cell 2B is a left upper molar, probably M^1^ (Fig 5A). It is two-rooted, with the anterior root being longer and more strongly developed than the posterior one. The tooth crown is heavily worn posterolingually but still retains a prominent paracone and metacone.

CGM 60570 from grid cell 4E is one-half of a large premolar (Fig 5B) with a crown so heavily worn that the two roots were severed. CGM 60570 retains just one of the two roots. The only enamel on the crown is a small patch on the less-worn side near the anterior or posterior end. We interpret this as the anterior half of a right P^4^, but it could also be the posterior half of a left P_4_. No meaningful measurements can be made of the remaining portion of the crown.

CGM 60568 from grid cell 3F is a large incisor with a long straight root and angled crown (Fig 5C). Much of the crown has been removed by a large area of very flat wear on its lingual side. This has removed not only the lingual side of the crown but much of the labial side. We interpret the tooth as a right I^1^. Here again, no meaningful measurements can be made of the remaining portion of the crown.

In the lower dentition, CGM 60558 from grid cell 2D is a lower molar with long roots (Fig 5D). One end of the crown has the characteristic notch marking the anterior surface. Based on size and tooth wear, we interpret this as a right M_2_.

CGM 60554 from grid cell 2D is another heavily worn half premolar (Fig 5E). In this case the crown preserves the two posterior cusps, and the most posterior of these is bordered by smaller labial and lingual denticles on a posterior cingulid. The posterior root is massive, measuring 40 mm in anteroposterior length and 28 mm in transverse width near the base of the crown. The width of the crown was approximately the same as the width of the root. This is almost certainly the posterior half of the right P_4_.

CGM 60579 from grid cell 4E/F is a relatively little worn left C_1_ (Fig 5F). This has a massive root that is mediolaterally flattened, slightly convex anteriorly at the level of the crown, and slightly concave posteriorly near the end of the root. The anteroposterior length and transverse width of the crown cannot be measured accurately, but visual comparison to C_1_ in CGM 42195 indicates that both teeth were about the same size (the latter has a crown 36.1 mm long and 25.4 mm wide). The crown of CGM 60579 measures 44 mm in height on the lingual side, with several millimeters missing due to apical wear. CGM 60579 was complete when found, but following recovery it was then subjected to high summer humidity causing salts within the dentine to swell and break. The breakage separated the crown from the root. These have since been reunited, but some intervening dentine could not be repaired and the crown is now no longer oriented properly relative to the root.

CGM 60561 from grid cell 3C is a well preserved right lower incisor (Fig 5G). It resembles the canine CGM 60579, but the crown and root are smaller and the posterior margin of the root has more of a sigmoid lateral profile. There is a well-defined, deep, oblique wear facet crossing the posterolateral part of the crown. Taken together, the size, robustness, and wear of the tooth indicate that it is a right I_2_.

CGM 60565 from grid cell 3E has a crown with all enamel worn away (Fig 5H). Its anterior and posterior margins are smoothly convex and concave, respectively. This is the most slender of all of the *Basilosaurus isis* incisors in WH 10001, and it narrows continuously to the end of its root. We interpret this as a right I_1_ with the medial side of the crown spalled away, but it could possibly be I_1_ from the left side.

#### Tooth wear of *Basilosaurus isis*

Two hallmarks of the permanent dentition in adult specimens of *Basilosaurus isis* are broken teeth and uneven occlusal tooth wear. The adult specimen CGM 42195 (Fig 1), for example, has maxillary teeth, P^2^ excepted, heavily worn on the left side, and maxillary teeth, C^1^ and P^1^ excepted, little worn on the right side. In the left mandible the C_1_ is broken off at the level of the alveolus, P_2_ is little worn, P_3_ is heavily worn, and adjacent P_4_ is moderately worn. In the right mandible C_1_ has light apical wear similar to that seen in CGM 60579 here, P_2_ and P_3_ are almost unworn, and P_4_ is so heavily worn that the anterior and posterior roots are almost separated. M_1_ is heavily worn on both sides, while M_2_ and M_3_ are little worn.

Permanent teeth of *B*. *isis* associated with WH 10001 are generally heavily worn, but they have similarly uneven occlusal tooth wear. Some premolars have their crowns worn through, separating the roots (CGM 60570 and 60554; Fig 5B and 5E), while other teeth like the left C_1_ (CGM 80579; Fig 5F) are little worn. The presence of heavy occlusal wear, especially on the premolars and molars, corroborate evidence from fusion of vertebral epiphyses that the WH 10001 *B*. *isis* specimen was adult and possibly even an old adult when it died.

### Dorudon atrox

**Fig 6:**
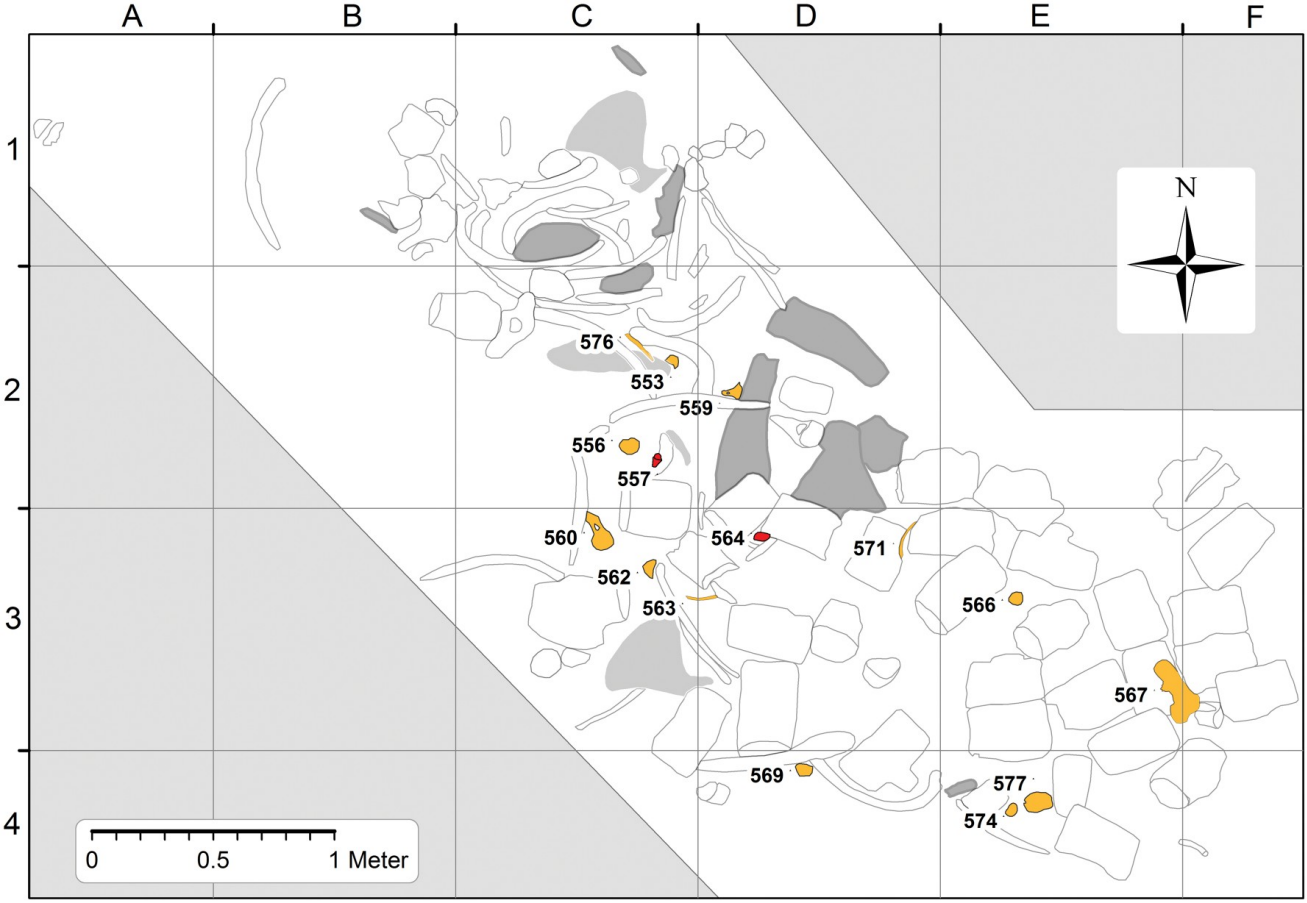
Map of *Basilosaurus isis* WH 10001 with locations of other vertebrate specimens superimposed. Juvenile *D*. *atrox* specimens, identified by the last three digits of their CGM museum number (CGM 60553, etc.), are illustrated in Figs 7 and 8. *Pycnodus mokattamensis* (CGM 60564) and *Carcharocles sokolowi* (CGM 60557) specimens are illustrated in Fig 9. Note the wide scatter of *D*. *atrox* remains, similar to the scatter of *B*. *isis* permanent teeth shown in Fig 4. Outlines of *B*. *isis* cranial remains are filled with darker shading, and outlines of *B*. *isis* forelimb elements are filled with lighter shading.

**Fig 7:**
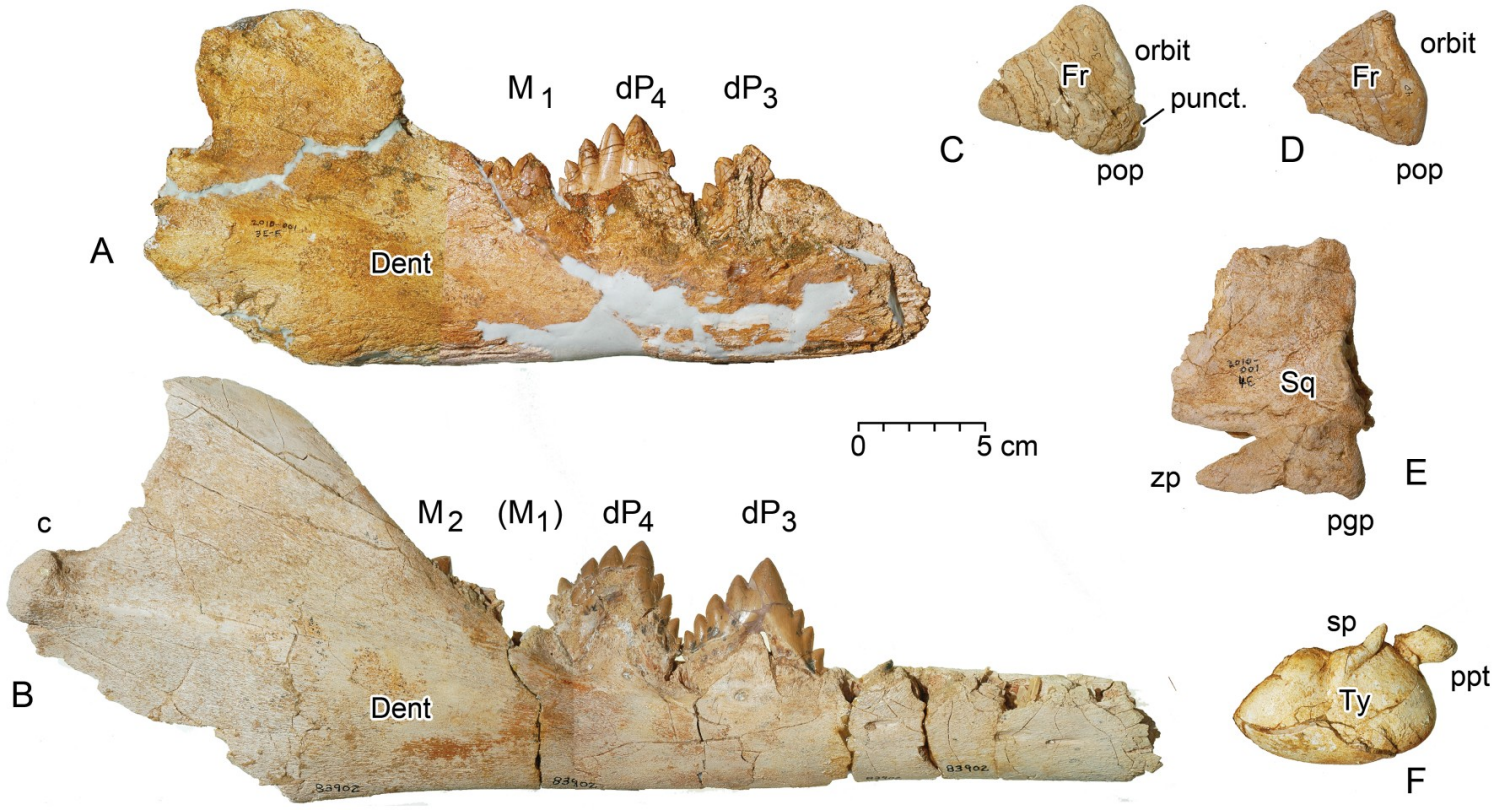
Cranial remains of juvenile *Dorudon atrox*. The position of each element in the WH 10001 *B*. *isis* excavation is shown in Fig 6, identified by the last three digits of its CGM number. A, CGM 60567, right dentary with crowns of deciduous milk teeth dP_3–4_ in place and the crown of M_1_ beginning to erupt (specimen is in ontogenetic stage 2 of 13 tooth eruption and dental wear stages of Uhen [29]). B, UM 83902, right dentary with alveoli for anterior premolars, crowns of dP_3–4_ in place, roots of M_1_, and the crown of M_2_ beginning to erupt. Uhen [29] classified this specimen, from the overlying Birket Qarun Formation in Wadi Al-Hitan, in his ontogenetic stage 4). C, CGM 60562, supraorbital process of a right frontal. D, CGM 60569, supraorbital process of a second slightly smaller right frontal. E, CGM 60577, right squamosal. F, CGM 60574, left tympanic bulla. Note that the CGM 60567 dentary is missing the anterior portion and mandibular condyle, and all of the other cranial elements show evidence of breakage or disarticulation. Abbreviations: *c*, mandibular condyle; *Dent*, dentary; *dP*_*3*_*-dP*_*4*_, lower deciduous premolar 3–4; *Fr*, frontal; *M*_*1*-2_, lower molar 1–2; *pgp*, postglenoid process; pop, postorbital process; *ppt*, posterior process of tympanic; *punct*, puncture bite mark on frontal; *sp*, sigmoid process; *Sq*, squamosal; *Ty*, tympanic; *zp*, zygomatic process.

**Fig 8:**
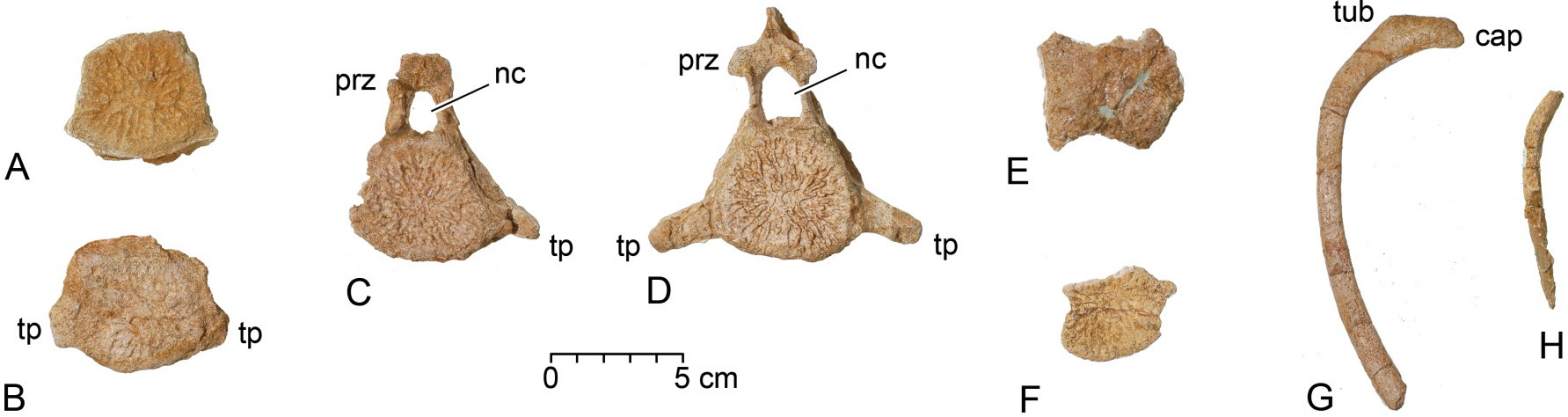
Postcranial remains of juvenile *Dorudon atrox*. The position of each element in the WH 10001 *B*. *isis* excavation is shown in Fig 6, identified by the last three digits of its CGM number. A, CGM 60553, centrum of a posterior thoracic vertebra. B, CGM 60578, centrum of a posterior thoracic vertebra. C, CGM 60559, posterior thoracic vertebra. D, CGM 60560, posterior thoracic vertebra. E, CGM 60566, possible centrum of a thoracic vertebra. F, CGM 60556, vertebral epiphysis. G, CGM 60571, left anterior rib. H, CGM 60576, midshaft of a rib of unknown position. Note that all vertebral centra all lack epiphyses. Abbreviations: *cap*, rib capitulum; *nc*, neural canal; *prz*, prezygapophysis; *tp*, transverse process; *tub*, rib tuberculum.

The distribution of *Dorudon atrox* specimens in the WH 10001 *Basilosaurus* excavation is illustrated in Fig 6, where 13 skeletal elements of *Dorudon* are mapped. All are labeled using the last three digits of their CGM museum number. *Dorudon* specimens include cranial and postcranial elements (Figs 7 and 8). Some *D*. *atrox* specimens are in or near the lower thorax of *Basilosaurus*, where stomach contents might be expected, but the rest are south and east of this, which is again consistent with movement by a northwest-to-southeast bottom current.

#### Dentary and teeth of *Dorudon atrox*

CGM 60567 (Fig 7A) is the most distinctive and informative specimen of *Dorudon atrox* found in the WH 10001 *Basilosaurus* excavation. It was found in grid cell 3E/F. CGM 60567 is a right dentary with dP_3_ and dP_4_ in place in the jaw, and M_1_ beginning to erupt behind dP_4_. The dentary is broken anteriorly at the position of dP_2_, and it is broken posteriorly just behind the ascending ramus so the mandibular condyle is missing. The only tooth that can be measured is dP_4_, and here only the length can be measured. The length (Table 2) is almost exactly the median (56.1 mm) for nine *D*. *atrox* dP_4_ lengths published in appendix 1B by Uhen [29].

CGM 60567 is similar to a more complete dentary of *D*. *atrox* collected from the overlying Birket Qarun Formation (UM 83902; Fig 7B), which is at a slightly later stage of tooth development. UM 83902 is in stage 4 of Uhen’s 13 age classes for *Dorudon* [29]. CGM 60567 on the other hand is in or near Uhen’s stage 2 of ontogeny. No dentaries of *D*. *atrox* have been collected representing any younger stage of ontogeny.

#### Cranial bones of *Dorudon atrox*

Four cranial elements of *Dorudon atrox* have been found in addition to the dentary. These include a left squamosal, a left tympanic bulla, and the supraorbital processes of two right frontal bones, the latter indicating the presence of at least two different *D*. *atrox* specimens.

CGM 60577 from grid cell 4E is a complete left squamosal of *D*. *atrox* (Fig 7E), which is amalgamated to a juvenile vertebral disc that is likewise referable to a *D*. *atrox* specimen. This has an open suture on the ventral surface of the zygomatic process for articulation with the jugal. It has open sutures anteriorly and dorsally for articulation with the left parietal, and it has open sutures posteriorly for articulation with the supraoccipital, exoccipital, and posterior process of the left periotic. Uhen [29] described a cranium, UM 100142, at this stage of squamosal fusion. It lacks teeth but he regarded it as probably younger than stage 1 in his sequence of *Dorudon* stages of ontogeny based on dental eruption.

CGM 60574 from grid cell 4E is a complete left tympanic bulla of *Dorudon atrox* (Fig 7F). The tympanic was found adjacent to a mass of flattened bone that could possibly be a *Basilosaurus* vertebra (other vertebrae of *Basilosaurus* here are not flattened), or it could possibly be part of a *Dorudon* cranium. The tympanic is partly obscured by matrix, but much of the lateral, ventral, medial, and posterior surfaces have been cleaned and exposed. The involucrum is thick and undeformed, however the outer lip of the bulla has collapsed into the tympanic cavity. The sigmoid process is prominent on the lateral surface of the bulla, as is the posterior process of the tympanic for articulation with the posterior process of the periotic. The anteroposterior length of the CGM 60574 bullar portion of the tympanic is 79 mm and the transverse width is approximately 50 mm. Uhen [29] gives ranges of 79.9–86.1 and 53.0–59.1 for these measurements in *D*. *atrox*. The CGM 60574 bulla is just below the small end of both ranges, which is not surprising for a juvenile. For comparison, the tympanic bulla of a female *Basilosaurus isis*, CGM 42195, measures 86 mm in anteroposterior length and 69 mm in transverse width.

CGM 60569 from grid cell 4D is the supraorbital portion of a right frontal of *Dorudon atrox* (Fig 7D). It is triangular in shape, with a slightly convex dorsal surface, and a dorsoventrally thin and slightly sigmoid finished edge of bone along its lateral and orbital margin anterior to the postorbital process (the sigmoid shape of the lateral margin is visible in Fig 7D). CGM 60569 has a dorsoventrally thick finished edge of bone sloping into the temporal opening on the posteromedial margin, medial to the postorbital process, and it has a cross section of laminar bone on the anteromedial side where it was broken from the remainder of the frontal shield. On the ventral side, the surface above the orbit is shallowly concave, and the surface above the temporal opening is sloping but relatively flat. The longest edge of the supraorbital triangle is the posteromedial edge, which is 55 mm in length.

CGM 60562 from grid cell 3C is the supraorbital portion of a second right frontal of *Dorudon atrox* (Fig 7C). It is similar to CGM 60569, with similar finished and broken edges, but CGM 60562 is slightly larger, measuring 67 mm along its posteromedial edge, and it thus resembles the dimensions in UM 100139, which represents a young individual of *D*. *atrox* with an erupting M^1^ [29]. CGM 60562 is conspicuous in having a distinct conical puncture in the most lateral surface of the postorbital process. This depression is oval in shape, measuring about 8 mm along an oblique anteroposterior axis and about 7.4 mm along a dorsoposterior axis. The depression is at least 8 mm deep. It mostly resembles the single bite mark on the frontal shield of UM 100139 in shape and outline and hence is interpreted here as representing a similar bite mark. Two additional possible bite marks are found on the dorsal and ventral sides of CGM 60562. The ventral one is located close to the broken anterior margin and about 14 mm from the lateral margin. This mark is small and circular, measuring about 5 mm in diameters. It is most similar to three bite marks found on UM 94814. The dorsal one reflects a rather indistinct 6 mm long, shallow depression. Its elongated outline in a more or less anteroposterior direction differs from the bite marks described above.

#### Postcranial bones of *Dorudon atrox*

Eight postcranial specimens of *Dorudon atrox* have been identified in addition to the cranial elements. These include four or possibly five vertebrae with centra, and parts of two ribs. Centrum measurements are given in Table 3.

**Table 3:** Measurements of *Dorudon atrox* vertebral centra recovered from the WH 10001 *Basilosaurus isis* excavation in Wadi Al-Hitan, Egypt.

Species	Grid cell	CGM specimen	Description	Antero-posterior length (mm)	Dorso-ventral height (mm)	Transverse width (mm)	Illustration
*Dorudon atrox*	2C	60553	Vertebral centrum	17*	48	—	Fig 8A
*Dorudon atrox*	2C	60556	Vertebral epiphysis	5	32	42	Fig 8F
*Dorudon atrox*	2D	60559	Vertebral centrum (T16?)	19*	48	56*	Fig 8C
*Dorudon atrox*	2/3C	60560	Vertebral centrum (T17?)	19*	50	54*	Fig 8D
*Dorudon atrox*	3E	60566	Vertebral centrum (?)	—	—	—	Fig 8E
*Dorudon atrox*	4E	60578	Vertebral centrum (T14 or 15?)	16*	46	56	Fig 8B

*Measurements with asterisks are estimated.

CGM 60553 from grid cell 2C is the centrum of a posterior thoracic vertebra of *D*. *atrox* (Fig 8A). The centrum is relatively short anteroposteriorly, and almost circular in cross section. Both epiphyses are missing, as are the neural arch and rib articulations.

CGM 60578 from grid cell 4E is the centrum of a posterior thoracic vertebra of *D*. *atrox* (Fig 8B). The centrum is again relatively short anteroposteriorly, and almost circular in cross section. Both epiphyses are missing, as is the neural arch. Short transverse processes with flat distal surfaces are present. CGM 60578 compares well with adult thoracics T14 or T15 in terms of transverse process development, as illustrated by Uhen [29]. The centrum measures 68 mm wide across the transverse processes. It remains attached to the inner surface of the CGM 60577 squamosal.

CGM 60559 from grid cell 2D is a posterior thoracic vertebra of *D*. *atrox* (Fig 8C). The centrum is similar to that of CGM 60553 and CGM 60578, but here a tall, narrow, neural arch is present with small but distinct prezygapophyses. The transverse process is complete on the left side, and this is longer than that of CGM 60578. CGM 60559 compares well with the adult thoracic T16 in terms of transverse process development [29]. Reconstructing the total width by estimation from the one complete transverse process, the vertebra appears to have been about 88 mm wide across the two transverse processes. The neural canal is 11 mm wide and 17 mm high.

CGM 60560 from grid cell 2/3C is the most complete posterior thoracic of *D*. *atrox* (Fig 8D). The centrum lacks epiphyses, but the neural arch and transverse processes are intact. Pre- and postzygapophyses are well developed, arising near the top of the neural arch. The base of the neural spine is present and the spine itself may be almost complete. CGM 60560 compares well with the adult thoracic T17 in terms of transverse process development [29]. The specimen measures 104 mm wide across the transverse processes. The neural canal is 15 mm wide and 23 mm high.

CGM 60566 from grid cell 3E is the right size to be an eroded centrum of *D*. *atrox* (Fig 8E), but it does not preserve enough morphology to measure reliably or to be certain of this attribution.

CGM 60556 from grid cell 2C is a vertebral epiphysis of *D*. *atrox* (Fig 8F). It is not complete, but its size and shape suggest that it is the epiphysis of an anterior thoracic vertebra.

CGM 60571 from grid cell 3D is a nearly complete left anterior rib of *D*. *atrox* (Fig 8G). This has the head and tuberculum well separated (ca. 28 mm), and a relatively long body. It is identified as a left rib because the neck near the tuberculum is slightly concave on one side and slightly convex on the other: the concave side is anterior. The total length of the rib as preserved is 151 mm, and it measures 7 mm in anteroposterior diameter and 9 mm in transverse diameter at midshaft. Part of the distal end may be missing.

CGM 60576 from grid cell 2C is part of the midshaft of a rib of *D*. *atrox* (Fig 8H). This rib, of unknown position, measures 7 x 10 mm in cross-sectional diameter.

### Pycnodus mokattamensis

Two specimens of *Pycnodus mokattamensis* were found in the WH 10001 *Basilosaurus* excavation. One specimen, a single tooth, was picked up on the surface the day that WH 10001 was discovered, before excavation began and before the mapping grid was constructed. The other more complete specimen is shown on the map in Fig 6, labeled with the last three digits of its CGM museum number (564). Anatomical terminology follows [44].

**Fig 9:**
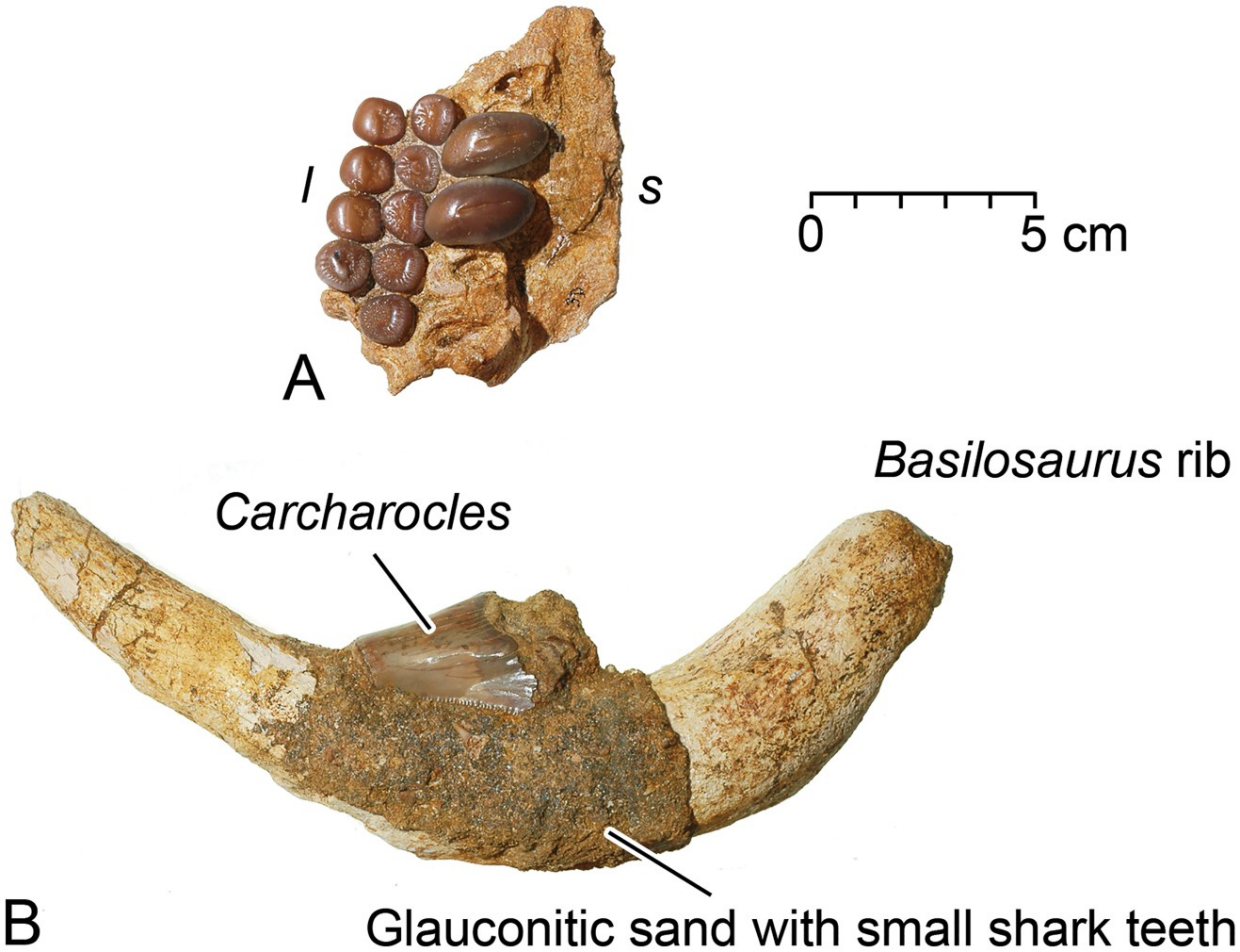
Remains of large fishes associated with WH 10001 *Basilosaurus isis*. A, left prearticular and crushing pavement-toothed lower dentition of the mollusk-eating bony fish *Pycnodus mokattamensis*. B, tooth of the large predatory lamniform shark *Carcharocles sokolowi* attached to a rib of *B*. *isis*. Abbreviations: *l*, lateral; *s*, symphyseal.

CGM 60564 from grid cell 3D preserves much of the left prearticular and left lower or prearticular dentition of *P*. *mokattamensis* (Fig 9A). The prearticular bone has the sutured medial or symphyseal edge well preserved. There is a toothless area on the prearticular paralleling the symphysis, and then three anteroposteriorly aligned rows of crushing teeth lateral to the toothless area. The dentition includes a medial row of large elliptical teeth with convex crowns, and two lateral rows of smaller, flatter, circular teeth. Tooth crowns in the medial row have a transverse central sulcus surrounded by polished enameloid and then radially crenulated enameloid. The medial edges of the crowns are positioned slightly anterior to their lateral edges, and the long axis of each medial tooth is oriented at an angle of about 80° relative to the symphyseal edge. CGM 60564 preserves two teeth in the medial row, the second and third (from front to back) of an original five or more medial teeth. Teeth in the lateral rows have a central depression surrounded by polished enameloid and then radially crenulated enameloid. There are five teeth present in the first lateral row, and four teeth in the second lateral row.

The prearticular of CGM 60564, as preserved, is 76 mm long anteroposteriorly, and 67 mm wide at its widest point. The second and third medial teeth of CGM 60564 measure 24.3 and 25.4 mm along the long axis of the crown, and 14.0 and 15.0 mm, respectively, perpendicular to this. The five teeth in the first lateral row average 11.1 mm in anteroposterior length and 11.5 mm in transverse width. The four teeth in the second lateral row average 10.8 mm in length and 12.4 mm in width.

CGM 60580 is a single tooth of *P*. *mokattamensis* from an unknown grid cell in the WH 10001 excavation. It is virtually identical to one of the medial-row prearticular teeth just described, and almost certainly comes from the left side. It could be one of the medial teeth missing from the CGM 60564 prearticular, but it does not fit any open alveolus in the latter so any break must have happened before burial.

The species *Pycnodus mokattamensis* was named by Priem [45] based on an upper or vomerine dentition with medial teeth 10 mm in anteroposterior length and 14–15 mm in transverse width, and lateral teeth 11–12 mm in length and 8–9 mm in width. [45] gives measurements of 13 x 7 mm for two lower teeth. Priem’s specimen is probably Bartonian (late middle Eocene) in age [18, 46]. CGM 60564 is a little larger than Priem’s specimen [45], and geologically younger in being Priabonian late Eocene in age.

### Carcharocles sokolowi

CGM 60557 from grid cell 2C is a single worn anterior tooth of the large shark *Carcharocles sokolowi* (Fig 9B). It was found attached to a *B*. *isis* rib in a thin mantle of glauconitic sand full of small shark teeth. The *Carcharocles* tooth has some apical wear and the base is badly eroded. We estimate that the crown was approximately 57 mm high (including the base) with a base estimated to have been 45 mm wide. It was probably shed by a scavenger following death of the WH 10001 *Basilosaurus* (see below), but it could possibly be part of an animal the *Basilosaurus* ingested.

## Discussion

The WH 10001 *Basilosaurus* excavation preserves evidence of four large vertebrate species found together: (1) a single adult skeleton of the large archaeocete *Basilosaurus isis* (Figs 3 and 4), with no duplication of parts indicating more than one individual; (2) thirteen skeletal elements of the medium-sized archaeocete *Dorudon atrox* (Fig 6), with duplicated supraorbital portions of right frontals indicating that a minimum of two juvenile skeletons are present; (3) the prearticular dentition of at least one pycnodontid bony fish *Pycnodus mokattamensis*; and (4) one tooth of the large lamnid shark *Carcharocles sokolowi*. *Basilosaurus*, *Dorudon*, and *Pycnodus* were all extinct by the end of the Eocene; lamnid sharks survive today.

To put these species in perspective, an adult *Basilosaurus isis* was about 15–18 meters long in life (Fig 1). *B*. *isis* was a marine predator with pointed anterior teeth and narrow multicusped slicing cheek teeth. An adult *Dorudon atrox* reached a length of about 5 meters [29], and *D*. *atrox* was a smaller marine predator with teeth similar to those of *B*. *isis*. Skeletons of juvenile *D*. *atrox* indicate an animal about 1.5–2 meters in length. *Pycnodus mokattamensis* was a large deep-bodied fish [47, 48], and the length of the prearticular described here yields a body length estimate of about one meter [49]. *P*. *mokattamensis* had pavement-like crushing teeth and, where known, its stomach contents indicate a monospecific diet of bivalve mollusks [50]. Finally, crown height of the tooth of *Carcharocles sokolowi* indicates a lamnid shark with a body length of about 5 meters [51]. Lamnids are characteristically fast swimmers and heavily-built. We hypothesize that this fossil assemblage represents a *B*. *isis* specimen including stomach contents and that at least *Dorudon* and *Pycnodus* served as prey for *Basilosaurus*.

### Stomach contents

Animals often die with the remains of meals in their stomachs, and in favorable circumstances these remains can be identified and associated with their consumer. Wings [52] listed several criteria for identification of gastroliths in fossil vertebrates, and these are viable as criteria for stomach contents in general: (1) contents are found in association with the skeleton of an appropriate consumer; (2) contents are found in an anatomically correct position within the ribcage of the consumer; (3) contents are clustered and separated by distance from other such clusters; and (4) contents are found in a low-energy depositional setting in sediment finer in clast size than any stomach contents of interest. Bite marks are an additional fifth criterion for stomach contents not applicable to gastroliths.

The *Dorudon* elements described above are identified as stomach contents because of their fragmentary nature and association with the large predator *Basilosaurus isis*. It is also important that bones and teeth identified as stomach contents were found in or ‘downstream’ from the inferred position of the *Basilosaurus* rib cage, in a cluster in the sense of association with the larger skeleton, and in a low-energy fine-sediment setting where bones and teeth are generally rare. Finally, some *Dorudon* elements interpreted as stomach contents exhibit bite marks plausibly made by the predator.

As outlined above, the WH 10001 *Basilosaurus* specimen is taphonomically interpreted as a whale fall that came to rest in a low-energy depositional environment influenced by bottom currents that moved individual bones but not the skeleton as a whole. The *B*. *isis* skeleton is no longer articulated, but it is clearly separated from other skeletons in the area. This separation means that stomach contents are similarly separated and thereby clearly associated with the WH 10001 skeleton.

We would expect ingested food to remain in or just behind the rib cage when a whale-fall skeleton remains articulated, because cetaceans have three or sometimes four stomachs in the posterior thorax and anterior lumbus [53, 54, 55] within and just behind the rib cage. Stomach contents were found in this position in a Miocene cetotheriid [56]. However, studies on dinosaurs [52], extant amphibians [57], and cetaceans [58] indicate that stomach contents are not always confined to the rib cage, and stomach-content clusters are not always found in the same position for a given species. Stomach contents can be found throughout the entire body cavity as in the present case.

When a whale dies in the sea, what happens next depends on many factors. Some whales float when they die, but some do not [59]. Gases generated by decomposition build up in the abdominal cavity and these affect buoyancy [37, 38]. Scavengers affect the integrity of a whale corpse and may cause it to lose buoyancy. Whale carcasses are skeletonized within days in some environments, but may also remain intact for months in other environments [60, 61]. Disturbance of a skeleton on the sea floor depends on the relative size of the scavengers [62]. Disturbance and scattering of a skeleton means stomach contents are likely to be disturbed and scattered as well. We interpret the *B*. *isis* carcass and its stomach contents as having fallen to the sea floor with the stomach walls and body cavity still intact, so that the entire mass was deposited together on the sea-floor where it was then further disturbed.

Finally, we expect the focal animal at a site to be the largest and most complete at the site, because feeding involves comminution and ingestion of food that will fit within a stomach. For a predator like *Basilosaurus* we expect its prey to be smaller, and we expect its prey to be less complete. Consequently, we interpret the scattered juvenile *Dorudon atrox* remains associated with the WH 10001 *Basilosaurus isis* skeleton to represent stomach contents of the *Basilosaurus*. Juvenile *D*. *atrox* at 1-1/2 to 2 meters in length, would have been large enough to reward *Basilosaurus* predation and too large to swallow whole. All of the *D*. *atrox* remains at the WH 10001 site are disarticulated, and the larger specimens, including the CGM 60567 dentary and the CGM 60562 frontal, show evidence of being bitten or broken to smaller size.

The postorbital process of CGM 60562 deserves special attention. It shows significant bite marks on its posterolateral and ventral sides, which resemble bite marks on the juvenile crania of *Dorudon atrox* specimens UM 100139 and UM 94814 described by Uhen [29] and Fahlke [30]. The bite marks are similar in their dimension, shape and outline, and hence provide evidence of *Basilosaurus isis* preying on a juvenile *D*. *atrox*. This idea was tested in the bite mark analysis of Fahlke [30]. She interpreted a deep mark on UM 100139 as having been produced by a *B*. *isis* canine or incisor. The WH 10001 *B*. *isis* investigated here was a moderately old individual based on the heavily worn crowns of the premolars and molars. These teeth are not likely to have caused the specific pits in CGM 60562, but incisors and canines of WH 10001, which retain their enameled crowns and relatively sharp apices, would produce such deep circular to oval depressions.

In CGM 60562 and the *D*. *atrox* specimens studied by Uhen [29] and Fahlke [30], bite marks are found on the frontal shield. This is also true in a fourth specimen, UM 100142, which shows at least one large injury on the left side of the frontal (see Fig 3F in [29]). All of the *D*. *atrox* specimens with bite marks are juveniles. The CGM 60567 dentary in Fig 7A has the first molar erupting behind what would have been a full set of milk teeth. Toothed whales the size of *Dorudon* nurse their young for 1-1/2 to 2 years [63], and as such the *D*. *atrox* specimens in the WH 10001 excavation were clearly too young to feed as scavengers on a *Basilosaurus* carcass. *B*. *isis* seemingly fed preferentially on very young *D*. *atrox*, attacking them in the head [30], which was probably the fastest way to inflict a fatal injury.

Scavenging of juvenile *Dorudon atrox* by *Basilosaurus isis* is a possibility, but the long rostrum, pointed incisors, and sharp cheek teeth of *B*. *isis* suggest that it was a meat-eating predator rather than a bone-crushing scavenger. As such, we propose a predator-prey relationship between *B*. *isis* and *D*. *atrox*, which we additionally support by three arguments:

1. There is a relatively high proportion of juvenile *D*. *atrox* compared to adults found as fossils in Wadi Al Hitan. Something killed the juvenile *D*. *atrox*, and we consider very young and small-sized dorudons as perfect target prey for large *Basilosaurus*. The fact that whales are hunting whales is supported by ample evidence of apex-predators (orcas) in the modern ocean. Although generally hunting in groups, which is a contrast to the proposed hunting behavior of *Basilosaurus*, orcas are well known to attack and kill young cetaceans [7]. We conclude that this appears to be the case in Eocene Wadi Al Hitan as well.
2. Some *Dorudon* specimens bear bite marks made by *B*. *isis* [29, 30]. Although bite marks alone hardly allow one to distinguish between active predation and scavenging, most of the bite marks observed on dorudons are located on the head, more specifically, on the frontal. This indicates that the head was the preferred region for *Basilosaurus* attacks leading most efficiently to death [30]. Exactly the same situation is shown in Fig 2c in Collareta et al. [64] for the predatory giant megatooth shark *Carcharocles megalodon* that bit a diminutive baleen whale. If we assume *Basilosaurus* being a scavenger, we would expect that *Basilosaurus* preferencially fed on regions of the dorudon body other than the head, for example the tail or thoracic region. The latter is documented for another shark species, the Recent great white shark that, beyond preying on various pinnepeds, is also known to ordinarily scavenge on large whale carcasses [65].
3. Using again the great white shark for comparative purposes, it is less reported that this predator scavenges on significantly smaller targets, but, as already mentioned above, on large whale carrion [65]. Consequently, we would expect that *Basilosaurus* preferably, if at all, scavenged on comparably large, energy-rich carrion instead of juvenile dorudons.

Against this background, we also interpret the CGM prearticular and isolated tooth of *Pycnodus mokattamensis* as additional stomach contents and prey of *Basilosaurus*. *P*. *mokattamensis*, with a body length of 1 meter, was again large enough to reward *Basilosaurus* predation and too large to swallow whole. *P*. *mokattamensis* was a mollusk-feeder with crushing teeth, bivalves are dispersed in the surrounding sediments, and *P*. *mokattamensis* is unlikely to have fed as a scavenger on a *Basilosaurus* carcass.

Recovery of 1.5 to 2 meter long juveniles of *Dorudon atrox* and 1 meter long *Pycnodus mokattamensis* as stomach contents in *Basilosaurus isis* provides the first direct evidence of the diet of *Basilosaurus isis* and, at the same time, extends the diet of *Basilosaurus* as a genus well beyond the 50 cm long sharks and bony fishes recorded as stomach contents in *B*. *cetoides* [17].

### Postmortem scavenging of the *Basilosaurus* skeleton

In addition to aquatic transport, we identify scavenging as a mechanism to separate and disperse bones of the WH 10001 *Basilosaurus isis* and its stomach contents. Some of the bones of *B*. *isis*, especially ribs, reveal small, long and deep pits, which we interpret as tooth marks produced by small species of lamniform sharks. The potential bite mark on the dorsal side of the postorbital process in the *D*. *atrox* specimen CGM 60562 could also represent such a shark mark, because this depression differs in shape and outline from bite marks interpreted as having been made by *B*. *isis*. When a whale carcass sinks to the sea floor a wide array of smaller sharks take over much of the scavenging (see Fig 6 in [65]). The presence of many smaller sharks scavenging on the sea floor may explain the abundance of smaller shark teeth in the thin mantle of glauconitic sand found on ribs and elsewhere within the WH 10001 *Basilosaurus* excavation (Fig 9).

*Carcharocles sokolowi*, represented by one large tooth, is not likely to have been a victim of *Basilosaurus* predation because a living shark this large would be a formidable adversary. A good living model for Eocene *C*. *sokolowi* is the extant great white shark, *Carcharodon carcharias*, which is an opportunistic scavenger on floating whale carcasses [65]. Interaction with scavengers the size of *C*. *sokolowi* may explain how the WH 10001 *Basilosaurus* skeleton became disarticulated and scattered (Fig 3), following [64, 66] on predatory megatooth sharks specialized in hunting whales on the coastal shelves of warm oceans.

### Apex predators

Lions, tigers, and large bears are commonly cited as apex predators on land. Orcas and great white sharks are commonly cited as apex predators in the sea. Most are large and achieve their dominance preying on smaller relatives. The 15–18 meter long archaeocete *Basilosaurus isis* and its slightly larger relative *Basilosaurus cetoides* are the largest predators known in the late Eocene. *Basilosaurus* is not known to have preyed on contemporary full-grown 5-meter long *Dorudon atrox* nor on 5-meter long *Carcharodon sokolowi*, but attacking animals this size would have been possible. Large body size, wide geographic range, and demonstrated predation on juvenile *D*. *atrox*, taken together, mark *Basilosaurus* as an apex predator in oceans of the late Eocene.

When oceans cooled near the end of the Eocene some archaeocetes evolved into more modern baleen and toothed whales, while others, including *Basilosaurus*, became extinct. Later cetaceans filling the role of marine apex predators include the 14–18 m long late Miocene physeteroid *Livyatan melvillei* [67] with smaller late Miocene *Acrophyseter deinodon*, *A*. *robustus*, and *Zygophyseter varolai* [68, 69], and finally the 6–8 m long killer whale *Orcinus* living today [70, 71]. One way to put these predators in perspective is to compare them to the middle and late Cenozoic rise in maximum size of the filter-feeding guild of marine mysticete whales illustrated in [72].

**Fig 10:**
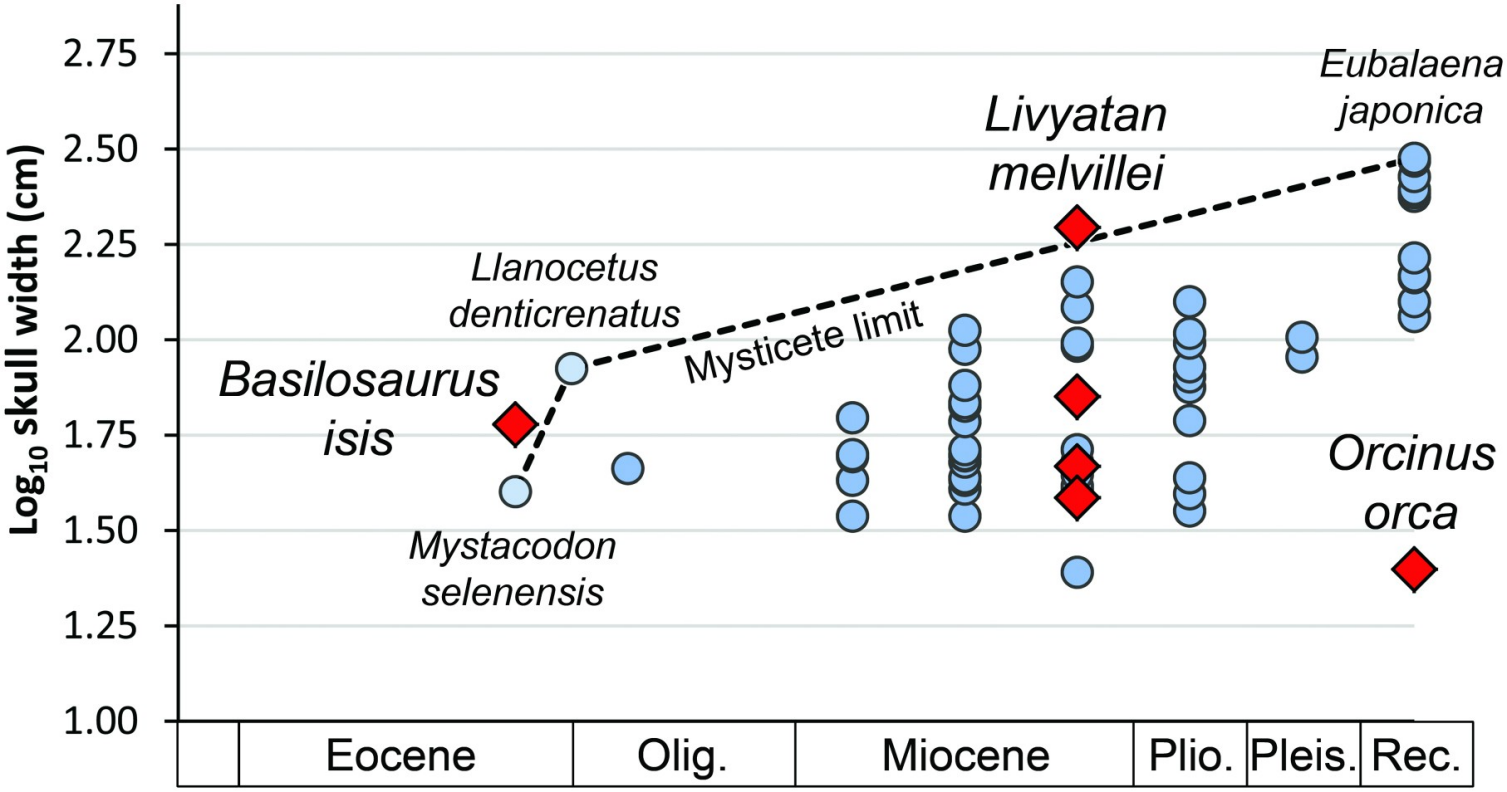
Skull widths as a size indicator for cetacean apex predators in relation to the size of contemporary baleen whales. For comparisons among whales in terms of their ability to feed on large prey, skull widths are shown with red diamonds for the late Eocene apex predator *Basilosaurus isis* (CGM 42195); late Miocene apex predators *Livyatan melvillei*, *Zygophyseter varolai*, *Acrophyseter deinodon*, and *A*. *robustus* (top down) [67, 69]; and extant apex predator *Orcinus orca* [70]. These are compared to the increasing limit (dashed line) for mysticete baleen whale sizes (blue circles) through Cenozoic time reported by [72]. Note that apex predators *B*. *isis* in the late Eocene and the physeteroid *L*. *melvillei* in the late Miocene are larger than contemporary mysticetes, but some late Miocene macroraptorial sperm whales (*Z*. *varolai*, *A*. *deinodon*, and *A*. *robustus*) and extant *O*. *orca* are smaller than most contemporary mysticetes. Large size is a common characteristic of apex predators, but large size is neither necessary nor sufficient: the largest mysticetes today are sometimes attacked by *O*. *orca* hunting in groups. Note large gaps in our understanding of the history of cetacean apex predators.

Eocene *Basilosaurus isis* evolved before the appearance of mysticetes, and it was larger than the seemingly-contemporary late Eocene mysticete *Mystacodon selenensis* (Fig 10). Several macroraptorial sperm whales lived in the late Miocene and the largest (in skull width), *Livyatan melvillei*, was larger than all mysticetes known from the Miocene. Extant *Orcinus orca* is an exception: an apex predator smaller than most contemporary mysticetes, but fully able to attack and kill the calves of one of the largest mysticetes known today, the humback whale [7]. The giant middle Miocene through Pliocene shark *Carcharocles megalodon*, with a body length estimated at 17–18 meters [51, 73], was probably comparable as an apex predator.

Late Eocene *Basilosaurus*, late Miocene *Livyatan*, and Pliocene to Recent *Orcinus* each has a known stratigraphic record that is relatively short, and little is known about whales as apex predators through the rest of Cenozoic time. Large consumers have ecological influence disproportionate to their abundance [72], and the size of marine predators deserves more attention than it has received.
